# Impact of a Symptom Checker App on Patient-Physician Interaction Among Self-Referred Walk-In Patients in the Emergency Department: Multicenter, Parallel-Group, Randomized, Controlled Trial

**DOI:** 10.2196/64028

**Published:** 2025-04-02

**Authors:** Malte L Schmieding, Marvin Kopka, Myrto Bolanaki, Hendrik Napierala, Maria B Altendorf, Doreen Kuschick, Sophie K Piper, Lennart Scatturin, Konrad Schmidt, Claudia Schorr, Alica Thissen, Cornelia Wäscher, Christoph Heintze, Martin Möckel, Felix Balzer, Anna Slagman

**Affiliations:** 1 Institute of Medical Informatics Charité – Universitätsmedizin Berlin, corporate member of Freie Universität Berlin and Humboldt-Universität zu Berlin Berlin Germany; 2 Division of Ergonomics Department of Psychology and Ergonomics Technische Universität Berlin Berlin Germany; 3 Emergency and Acute Medicine Campus Virchow-Klinikum and Campus Charité Mitte Charité – Universitätsmedizin Berlin, corporate member of Freie Universität Berlin and Humboldt-Universität zu Berlin Berlin Germany; 4 Institute of General Practice and Family Medicine Charité – Universitätsmedizin Berlin, corporate member of Freie Universität Berlin and Humboldt-Universität zu Berlin Berlin Germany; 5 Institute of Biometry and Clinical Epidemiology Charité – Universitätsmedizin Berlin, corporate member of Freie Universität Berlin and Humboldt-Universität Berlin Germany

**Keywords:** digital health, triage, symptom checker, patient-centered care, eHealth apps, mobile phone, decision support systems, consumer health information, health literacy, randomized controlled trials, null results, emergency care, patient-physician-interaction, patient satisfaction

## Abstract

**Background:**

Symptom checker apps (SCAs) are layperson-facing tools that advise on whether and where to seek care, or possible diagnoses. Previous research has primarily focused on evaluating the accuracy, safety, and usability of their recommendations. However, studies examining SCAs’ impact on clinical care, including the patient-physician interaction and satisfaction with care, remain scarce.

**Objective:**

This study aims to evaluate the effects of an SCA on satisfaction with the patient-physician interaction in acute care settings. Additionally, we examined its influence on patients’ anxiety and trust in the treating physician.

**Methods:**

This parallel-group, randomized controlled trial was conducted at 2 emergency departments of an academic medical center and an emergency practice in Berlin, Germany. Low-acuity patients seeking care at these sites were randomly assigned to either self-assess their health complaints using a widely available commercial SCA (Ada Health) before their first encounter with the treating physician or receive usual care. The primary endpoint was patients’ satisfaction with the patient-physician interaction, measured by the Patient Satisfaction Questionnaire (PSQ). The secondary outcomes were patients’ satisfaction with care, their anxiety levels, and physicians’ satisfaction with the patient-physician interaction. We used linear mixed models to assess the statistical significance of primary and secondary outcomes. Exploratory descriptive analyses examined patients’ and physicians’ perceptions of the SCA’s utility and the frequency of patients questioning their physician’s authority.

**Results:**

Between April 11, 2022, and January 25, 2023, we approached 665 patients. A total of 363 patients were included in the intention-to-treat analysis of the primary outcome (intervention: n=173, control: n=190). PSQ scores in the intervention group were similar to those in the control group (mean 78.5, SD 20.0 vs mean 80.8, SD 19.6; estimated difference –2.4, 95% CI –6.3 to 1.1, *P*=.24). Secondary outcomes, including patients’ and physicians’ satisfaction with care and patient anxiety, showed no significant group differences (all *P*>.05). Patients in the intervention group were more likely to report that the SCA had a beneficial (66/164, 40.2%) rather than a detrimental (3/164, 1.8%) impact on the patient-physician interaction, with most reporting no effect (95/164, 57.9%). Similar patterns were observed regarding the SCA’s perceived effect on care. In both groups, physicians rarely reported that their authority had been questioned by a patient (intervention: 2/188, 1.1%; control: 4/184, 2.2%). While physicians more often found the SCA helpful rather than unhelpful, the majority indicated it was neither helpful nor unhelpful for the encounter.

**Conclusions:**

We found no evidence that the SCA improved satisfaction with the patient-physician interaction or care in an acute care setting. By contrast, both patients and their treating physicians predominantly described the SCA’s impact as beneficial. Our study did not identify negative effects of SCA use commonly reported in the literature, such as increased anxiety or diminished trust in health care professionals.

**Trial Registration:**

German Clinical Trial Register DRKS00028598; https://drks.de/search/en/trial/DRKS00028598/entails

**International Registered Report Identifier (IRRID):**

RR2-10.1186/s13063-022-06688-w

## Introduction

It has become common for the general population to seek health-related information online. In the European Union, more than 1 in 2 citizens searched for health-related information online in the 3 months preceding the survey [1]. Similarly, 74.4% of US adults consulted the internet first when seeking health information during their most recent inquiry [2]. In a German panel study, a fifth of respondents identified the internet as their primary source of health information [3]. In particular, seeking health-related information online is common before consulting medical services among low-urgency acute care patients [4,5]. While qualitative studies highlight concerns from both patients [6-8] and health care professionals [9-12] regarding online information exacerbating patient anxiety [13] and undermining the patient-physician relationship, 2 quantitative observational studies investigating the effects of online information seeking showed positive effects on the perceived quality of care received and the patient-physician interaction [5,14]. However, results from an interventional study measuring these metrics as secondary outcomes did not provide evidence for such a positive effect [4].

One particular source of online health information is a symptom checker app (SCA). These consumer apps offer suggestions on potential diagnoses or an urgency assessment based on the self-reported signs and symptoms entered by users. SCAs face similar concerns to web-based health information–seeking behavior in general, as described above, particularly regarding the induction of anxiety [15-17]. Studies estimate that the proportion of SCA users in the German population ranges between 6% and 13% [16,18,19]. Some national health care services [20,21], health care systems [22,23], hospital networks [24], and insurance companies [25] have already integrated SCAs into their service pathways.

So far, research studies have primarily focused on assessing the accuracy and safety of SCA advice [26-38], their (potential) impact on patient journeys and resource allocation [28,39-43], and users’ experiences and expectations [6,7,17,30,38,41,44], but not on SCAs’ actual impact on patient-centered, clinically relevant outcomes. Four recent reviews concluded that more research on SCAs’ impacts in real-life settings is needed to assess their utility [45-48]. To address this research gap, we conducted a multicenter randomized controlled trial evaluating the effect of SCA usage in an acute care setting. We focused on patient-physician interaction, satisfaction with care, and users’ anxiety, as these areas frequently feature in discussions about the impact of SCAs and online health information on care [4,5,9,10,17,49]. We excluded questions regarding the SCA’s utility for improving patient allocation. The primary hypothesis was that the intervention would enhance patients’ satisfaction with their interaction with the treating physician. Secondary hypotheses included improvements in patients’ satisfaction with the health care received and their anxiety levels.

## Methods

### Study Design, Setting, and Participants

We conducted a multicenter, controlled parallel-group trial with balanced randomization at 3 study sites in Berlin, Germany. Two study sites were the emergency departments (EDs) of a large tertiary care university hospital: CCM (Charité – Universitätsmedizin Berlin, Campus Mitte in Berlin-Mitte) and CVK (Campus Virchow-Klinikum in Berlin-Wedding). Both EDs provide a wide spectrum of nonpediatric care, handling approximately 50,000 patient encounters annually, and operate an adjunct acute medical admissions ward. The third study site was an emergency practice operated by Berlin’s Association of Statutory Health Insurance Physicians (Kassenärztliche Vereinigung Berlin), located adjacent to the ED of a local hospital (Jüdisches Krankenhaus Berlin in Berlin-Gesundbrunnen [JKB]). This outpatient clinic provides urgent care outside regular office hours for approximately 4000 walk-in patients per year. It is typically staffed by 1 specialist physician and 1 medical assistant. The emergency practice serves self-referred patients who do not require treatment in the hospital-run ED located in the same building. This stratification of emergency care is designed to ensure that inpatient resources are reserved for urgently triaged patients. Key inclusion criteria were self-referred walk-in patients aged 18 years or older with sufficient German or English language proficiency, the ability to provide informed consent, and a treatment urgency rating of yellow, green, or blue according to the Manchester Triage System (ie, MTS 3-5, respectively) as assigned by the triage nurse. Exclusion criteria were patients treated without waiting time; patients whose chief complaint was already known to them; patients requiring isolation; patients unable to handle a tablet computer, as determined by either their own assessment or that of the study personnel; and patients who had already consulted an SCA for their current complaints before seeking care.

### Randomization and Masking

Participants were randomly assigned to either use the SCA before their first encounter with a treating physician or receive care as usual (1:1 ratio). Balanced block randomization with variable block lengths of 8, 10, and 12 was used. The recruiting study personnel were blinded to block size. Each trial site received its own allocation sequence, which was stored in sequentially numbered, opaque, and sealed envelopes. The allocation sequence was generated by the Institute of Medical Informatics using the R package blockrand (R Foundation) [50] by a researcher (MLS) who was not actively involved in recruitment. Allocation was concealed until the point of randomization, which occurred immediately after the patient consented to participate in the trial. Because of the nature of the intervention, participants, study personnel, and health care providers were not masked to group assignment after randomization.

### Procedures

Following initial triage, patients underwent eligibility screening conducted by study personnel (study nurse, student research assistant, or study physician). Upon providing informed consent, they were randomly assigned to either the control or intervention group (see details on randomization below). Before their first encounter with the treating physician, all participants completed a baseline survey assessing baseline anxiety, prior SCA use, and affinity for technology interaction [51]. A survey on sociodemographic and other variables that do not change during a patient’s stay—such as age, sex, native language, country of residence, level of education, net household income, frequency of internet, tablet, and smartphone use, self-perceived health and chronic morbidity [52], self-efficacy [53], and eHealth literacy [54]—was administered at a suitable time during their visit, either before or after their encounter with the treating physician (sociodemographic survey). Additionally, after seeing the treating physician—or in exceptional cases, within 72 hours thereafter—all participants were asked to complete a postencounter survey. Participants in the intervention group completed the self-assessment of their symptoms using the SCA after taking the baseline survey but before their first encounter with the treating physician. Following SCA use and before their consultation with a physician, they completed a post-SCA survey assessing their experience with the SCA and their level of anxiety. All surveys were administered via a tablet computer, with study personnel providing instructions on its use.

Study personnel ensured that a printout of the SCA summary report was available to treating physicians at the time of their first encounter with patients in the intervention group. However, to avoid interfering with the patient-physician relationship and care provision, study personnel neither encouraged nor discouraged physicians from engaging with the summary report. All participating physicians were aware that such reports would be available for patients in the intervention group. After providing care, the participating physicians completed a paper-based survey (physician-sided Patient Satisfaction Questionnaire [PSQ]), which assessed their satisfaction with the care provided, whether the patient questioned their authority, and their appraisal of the SCA’s helpfulness and impact on the patient encounter.

At the JKB trial site, all patients presenting on recruiting days were screened for eligibility. However, due to the higher patient volume at CCM and CVK, it was not feasible to screen all patients for eligibility at these sites.

The SCA used in the intervention (2024 Ada Health GmbH [55]) was developed independently of the researchers involved in this trial. We selected this particular commercial SCA from among the many available based on existing literature regarding its reported diagnostic and triage accuracy, the safety of its advice, its usability, the breadth of conditions and chief complaints covered [28,33,44,56], and its availability in both German and English. The SCA requires users to provide basic demographic information (such as age and sex), past medical history (including smoking behavior and prior diagnoses), and details about their current medical complaints. In this initial step, users can select an unlimited number of symptoms [57]. They then provide additional information by answering a series of closed questions with binary or multiple-choice answer options, presented by the SCA in a conversational format. These “conversations” typically last about 6-8 minutes [29,56,58]. The SCA then provides an assessment of the urgency of the complaints and suggests 1-5 probable causes (diagnostic suggestions), illustrated using a Sankey diagram. During the trial period, the SCA did not inquire about users’ intent to seek care, their own urgency assessment of their complaints, or the diagnoses they suspected. The SCA’s report also summarizes the findings that the user affirmed or denied. The SCA generates its suggestions using a medical knowledge base that hard-codes libraries of signs, symptoms, and diagnoses, along with their relationships, and applies a Bayesian network algorithm [59]. The company behind Ada Health describes this algorithm as “artificial intelligence” [60]. Further details of the SCA’s underlying algorithm are not publicly available. According to a 2024 study [16], Ada Health is the second most frequently used SCA in Germany. The developers also report that it is widely used in other countries, including Australia [61].

In the care-as-usual group, patients used the tablet only to complete the questionnaires (baseline, sociodemographic, and postencounter surveys) and did not have access to the SCA for self-assessing their symptoms. Patients in both groups were not prohibited from searching for online health information or using an SCA on their own devices during the study.

Study personnel closely observed patients for any signs of discomfort during the study procedures. In both groups, clinical routine care was conducted as usual. Study procedures were interrupted as needed for clinical interventions or other necessary reasons.

### Outcomes

#### Primary Outcome

The primary outcome was participants’ satisfaction with their interaction with the treating physician, assessed using the PSQ [62,63]. This instrument consists of visual analog scales ranging from 0 to 100 for each of the 5 items. A participant’s overall satisfaction is defined as the average score across all 5 items, with higher values indicating greater satisfaction. Participants’ responses on the primary outcome were collected in the postencounter survey before discharge or, in exceptional cases, within 72 hours of discharge if they left the trial site without completing the questionnaire. We considered a mean difference of 5 points (on a 0-100 scale) between treatment groups to be clinically relevant.

#### Secondary and Exploratory Outcomes

Participants’ satisfaction with the care received was measured using the 8-item Fragebogen zur Patientenzufriedenheit (ZUF-8) [64], the German version of the 8-item Client Satisfaction Questionnaire (CSQ-8) [65]. The ZUF-8 scale ranges from 8 to 32 points, with higher values indicating greater satisfaction. As some participants did not respond to all items of the ZUF-8 (each rated on a scale from 1 to 4), we calculated the average value of the items they answered. Therefore, we report our results for the ZUF-8 on a scale from 1 to 4. This deviates from the protocol, which did not include modifications to account for missing values. We expected a 1-point difference in care satisfaction between the intervention and control groups on the original ZUF-8 scale, corresponding to 0.125 points on the 1-4 scale.

Participants’ anxiety was measured up to 3 times during the trial: initially after recruitment (baseline), after using the SCA for participants in the intervention group, and finally after the patient-physician interaction. We used a visual analog scale ranging from 0 to 100, with higher values indicating greater anxiety [66]. Physicians’ satisfaction with the patient-physician interaction was measured using the physician version of the PSQ [62,63], which rephrases the PSQ items from the physician’s perspective.

As additional exploratory outcomes, we report participants’ perceived effect of the SCA on the patient-physician interaction and patient care (intervention group only; two 5-point Likert scales), the physicians’ assessment of the helpfulness of the SCA report (intervention group only; five 3-point Likert scales), and physicians’ satisfaction with the care they delivered, including overall satisfaction, time to diagnosis, and patient length of stay (measured on a visual analog scale ranging from 0 to 100).

### Sample Size Calculation

The trial group made a reasoned choice that a mean difference of at least 5 points in the PSQ score after the physician encounter between the intervention and control groups would be clinically relevant. In the literature, we found an SD of the patient-facing PSQ ranging from 14 to 17 points [63]. Assuming a standardized mean difference of 0.3 and equal variance, and considering a 2-sided α of .05, a power of 0.80, and a medium-sized dropout rate of 20%, a total of 440 patients were needed (ie, 220 in each trial arm). For the purpose of sample size calculation, we conservatively used a 2-sample (paired) *t* test for independent groups. However, our a priori planned analysis of the primary endpoint involves a mixed-effects model, which accounts for the clustered data structure (details below).

### Statistical Analyses

Descriptive statistics are presented as mean with SD, median with IQR, or frequency and proportion, depending on the scale and distribution.

We conducted our primary analysis based on the modified intention-to-treat principle, including all randomly assigned patients who provided responses to at least one item related to the respective primary or secondary outcome measure in the postencounter survey. Subgroup analyses by study site were conducted as preplanned.

We analyzed the primary outcome—patients’ satisfaction with the patient-physician interaction (measured by the PSQ)—using a linear mixed model with intervention as a fixed effect and study site as a random effect. Only participants who responded to at least one PSQ item were included. We report group means and SDs, the linear mixed model estimator, 95% CIs, and *P* values. Statistical analyses were conducted using R (version 4.4.0; R Foundation) [67], with data cleaning performed using tidyverse [68]. CIs were bootstrapped, and *P* values were calculated using the R packages lme4 [69] and parameters [70]. For secondary outcomes (patients’ satisfaction with the care received, change in anxiety levels after SCA use, the proportion of participants more anxious after the physician encounter than at baseline, and physicians’ satisfaction with patient-physician interaction), our analyses followed the same approach as for the primary outcome. As planned, we conducted no adjustments of *P* values due to multiplicity. To address missing data in primary and secondary outcomes, multiple imputation techniques were used as sensitivity analysis (see Table S3 in Multimedia Appendix 1). Regarding exploratory outcomes, we report descriptive statistics only.

The researchers statistically assessing the primary outcome were blinded to group assignment. Although not described in the previously published study protocol, we imputed missing data for primary and secondary endpoints as a sensitivity analysis using the R package mice [71].

### Ethical Considerations

All participants provided written informed consent. We made consent forms and participant-facing information leaflets on the study available in English and German. The information leaflet included information on the study’s intended purpose, design, procedure, the responsible persons, how and by whom their personal data were processed for the purposes of the study, and the participants’ rights including rights stemming from the European Union’s General Data Protection Regulation. The trial, the consent forms, and information leaflets were approved by the Institutional Review Board at Charité – Universitätsmedizin Berlin (reference number: EA2/284/21). We put in place technical and organizational measures to adhere to the European Union’s General Data Protection Regulation with the support of the Charité – Universitätsmedizin Berlin’s Clinical Trial Office. These measures included pseudonymizing data before conducting analyses, limiting access to all trial data, and storing and processing potentially reidentifiable trial data on servers owned and maintained by Charité – Universitätsmedizin Berlin. To render data reported in this paper anonymous, we only report aggregate statistics. The trial was prospectively registered in the German Clinical Trials Register (DRKS-ID: DRKS00028598). The protocol was previously published [72]. This manuscript follows the CONSORT (Consolidated Standards of Reporting Trials) statement [73]. Each participating patient received a €10 (US $10.8) gift voucher redeemable at over 300 brands and e-commerce platforms. Treating physicians received a €5 (US $5.42) gift voucher for each survey they completed on an encounter with a patient enrolled in the trial.

### Role of the Funding Source

The study funder had no role in the study design, data collection, data analysis, data interpretation, report writing, or the decision to submit for publication. Similarly, the developer of the SCA, Ada Health, was not a study sponsor and had no involvement in any of these aspects.

## Results

### Recruitment and Participant Characteristics

**Figure 1:**
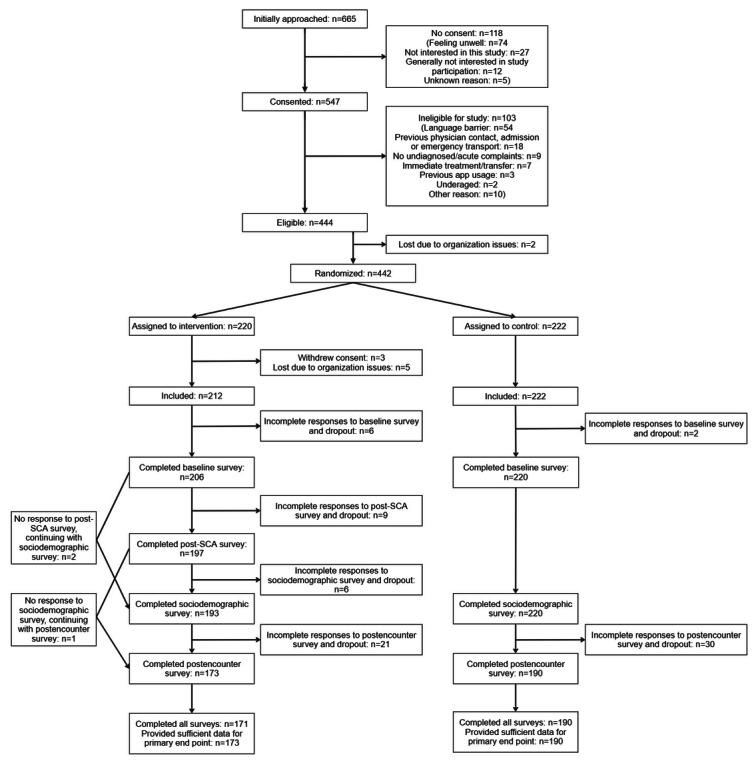
Trial profile outlining the total number of patients screened, randomized, completing study surveys at multiple time points during their stay, and included in the primary end point analysis. We conducted a multicenter, controlled, parallel-group trial at 3 study sites in Berlin, Germany. Between April 2022 and January 2023, we recruited self-referred adult walk-in patients presenting with an acute, undiagnosed chief complaint. The intervention group self-assessed their complaints using an SCA before their first encounter with a treating physician, while the control group received usual care. After the patient-physician encounter, we surveyed both patients and physicians on the patient-physician interaction and their satisfaction with care. SCA: symptom checker app.

Recruitment took place from April 11, 2022, to January 25, 2023. The recruitment phase was extended beyond the planned 6 months to accommodate staff shortages and COVID-19–related restrictions. Recruitment concluded upon reaching the predetermined sample size. A total of 442 participants were enrolled, with 220 randomly assigned to the intervention group and 222 to the control group. Two eligible participants in the control group dropped out immediately after randomization—due to organizational issues (n=1) and a medical condition (n=1). Three participants assigned to the intervention group withdrew their consent, and 5 additional participants were lost due to organizational issues. Of the 434 included participants, 188 (43.3%) identified as male, 220 (50.7%) as female, and 4 (0.9%) as diverse (with 22 participants not indicating their sex). The median age was 33 (IQR 26-45) years. Full details of group characteristics at baseline are provided in Table 1. The baseline survey was completed by 426 participants (206 in the intervention group and 220 in the control group). Of these, 363 provided sufficient data to assess the primary outcome (173 in the intervention group and 190 in the control group), with all but 2 (both in the intervention group) completing all surveys. Figure 1 presents the number of participants who completed the surveys over time.

Table S1 in Multimedia Appendix 1 compares the sex and age distributions of enrolled patients with those of all patients who presented at the trial sites during the study period. See Multimedia Appendix 2 for the CONSORT checklist.

**Table 1:** Baseline characteristics^a^.

Characteristics		Control (n=222)	Intervention (n=212)
Age (years), median (IQR)		36 (28-47)	30 (25-41)
**Sex, n (%)**			
	Male	96 (43.2)	92 (43.4)
	Female	122 (55.0)	98 (46.2)
	Other	2 (0.9)	2 (0.9)
	NA^b^	2 (0.9)	20 (9.4)
Questionnaire completed in English, n (%)		49 (22.1)	54 (25.5)
**Native language, n (%)**			
	German	120 (54.1)	103 (48.6)
	English	20 (9.0)	20 (9.4)
	Other	78 (35.1)	68 (32.1)
	NA	4 (1.8)	21 (9.9)
**Residence, n (%)**			
	Currently residing in Germany	209 (94.1)	175 (82.5)
	Currently residing outside of Germany	10 (4.5)	16 (7.5)
	NA	3 (1.4)	21 (9.9)
**Education, n (%)**			
	Student	6 (2.7)	7 (3.3)
	Basic education	1 (0.5)	0 (0)
	Lower secondary education after year 9 or 10	13 (5.9)	12 (5.7)
	Lower secondary education until year 10	38 (17.1)	25 (11.8)
	High school degree for university entrance	135 (60.8)	124 (58.5)
	Other school leaving certificate (eg, awarded abroad)	21 (9.5)	23 (10.8)
	NA	8 (3.6)	21 (9.9)
**Professional qualification, n (%)**			
	No qualification; still undergoing professional training (eg, student, trainee, in prevocational training, intern)	28 (12.6)	26 (12.3)
	No professional qualification and not undergoing training	12 (5.4)	13 (6.1)
	Apprenticeship (receiving vocational training from a company)	21 (9.5)	10 (4.7)
	Training at a vocational school or a commercial school (combined vocational and academic education)	32 (14.4)	17 (8.0)
	Technical college (eg, guild school, school for technicians, or vocational or professional academy)	14 (6.3)	11 (5.2)
	University of applied sciences, engineering college, advanced technical college	18 (8.1)	16 (7.5)
	University or college	82 (36.9)	87 (41.0)
	Other formal qualification (eg, acquired abroad)	5 (2.3)	8 (3.8)
	NA	10 (4.5)	24 (11.3)
**Net household income^c^, n (%)**			
	Less than €1800	53 (23.9)	61 (28.8)
	€1801 to €2800	50 (22.5)	41 (19.3)
	€2801€ to €4000	44 (19.8)	25 (11.8)
	More than €4000	39 (17.6)	43 (20.3)
	NA	36 (16.2)	42 (19.8)
Anxiety (0-100), median (IQR)		73 (60-86)	72 (60-80)
**Triage category, n (%)**			
	3	85 (38.3)	67 (31.6)
	4	104 (46.8)	110 (51.9)
	5	3 (1.4)	1 (0.5)
	NA	30 (13.5)	34 (16.0)
Self-efficacy (ASKU^d^), median (IQR)		4.0 (3.3-4.3)	4.0 (3.3-4.3)
eHealth literacy (eHEALS^e^), median (IQR)		3.5 (3.0-3.9)	3.6 (3.1-4.1)
**Self-perceived health, n (%)**			
	Very good	38 (17.1)	43 (20.3)
	Good	110 (49.5)	83 (39.2)
	Fair	54 (24.3)	47 (22.2)
	Bad	14 (6.3)	18 (8.5)
	Very bad	4 (1.8)	1 (0.5)
	NA	2 (0.9)	20 (9.4)
**Chronic morbidity, n (%)**			
	Yes	99 (44.6)	74 (34.9)
	No	115 (51.8)	115 (54.2)
	NA	8 (3.6)	23 (10.8)
**Internet usage, n (%)**			
	Daily	201 (90.5)	178 (84.0)
	Several times a week	15 (6.8)	9 (4.2)
	Several times a month	4 (1.8)	2 (0.9)
	Never	0 (0)	3 (1.4)
	NA	2 (0.9)	20 (9.4)
**Tablet usage, n (%)**			
	Daily	37 (16.7)	33 (15.6)
	Several times a week	32 (14.4)	22 (10.4)
	Several times a month	24 (10.8)	21 (9.9)
	Several times a year	36 (16.2)	31 (14.6)
	Never	86 (38.7)	83 (39.2)
	NA	7 (3.2)	22 (10.4)
Previously used SCAs^f^		12 (5.4)	14 (6.6)

^a^We conducted a multicenter, controlled parallel-group trial at 3 study sites in Berlin, Germany. Between April 2022 and January 2023, we recruited self-referred adult walk-in patients presenting with an acute, undiagnosed chief complaint. The intervention group self-assessed their complaints using an SCA before their first encounter with a treating physician, while the control group received care as usual. After the patient-physician encounter, we surveyed both patients and physicians regarding the patient-physician interaction and their satisfaction with care.

^b^NA refers to both missing responses and respondents who indicate their preference not to provide a response. The higher dropout rate in the intervention group yields a greater proportion of NAs relative to the control group.

^c^€1=US $1.08.

^d^ASKU: Allgemeine Selbstwirksamkeit Kurzskala.

^e^eHEALS: eHealth Literacy Scale.

^f^SCA: symptom checker app.

### Primary Outcome

Across all 3 recruitment sites, participants’ mean PSQ scores were close to 80 (on a scale from 0 to 100) in both groups (Table 2). The linear mixed-effects model showed no significant fixed effect for the group (estimate –2.4, 95% CI –6.3 to 1.1, reference: control group, *P*=.24). While patient-reported PSQ scores were similar between the control and intervention groups at 2 recruitment sites (CCM and JKB), participants from the CVK site in the intervention group reported, on average, 11 points lower satisfaction with the patient-physician interaction compared with their controls (see Table S2 in Multimedia Appendix 1). The use of different imputation methods showed no significant effects of missing data (see Table S3 in Multimedia Appendix 1).

**Table 2:** Primary and secondary endpoints according to each study group in the modified intention-to-treat population^a^.

Endpoints		Control	Intervention	*P* value
**Patient satisfaction with patient-physician interaction (patient-sided PSQ^b^)**				.24
	Descriptive, mean (SD); n	80.8 (19.6); 190	78.5 (20); 173	
	Estimate for the fixed effect of the study group in the linear mixed model (intervention to control group), 95% CI	N/A^c^	–2.4 (–6.3 to 1.1)	
**Patient satisfaction with care (ZUF-8^d^)**				.27
	Descriptive, mean (SD); n	2.6 (0.2); 190	2.6 (0.2); 173	
	Estimate for the fixed effect of the study group in the linear mixed model (intervention to control group), 95% CI	N/A	0.02 (–0.02 to 0.06)	
**Change in anxiety level, before SCA^e^ use to after**				.96
	Descriptive, mean (SD); n	N/A	–1.7 (13.5); 199	
	Estimate for the fixed effect of the study group in the linear mixed model, 95% CI	N/A	–0.1 (–5.0 to 4.5)	
**Participants more anxious after the physician encounter than at baseline**				.93
	n/N (%)	39/191 (20.4)	36/173 (20.8)	
	Estimate for the fixed effect of the study group in the generalized linear mixed model, 95% CI	N/A	0.0 (–0.5 to 0.6)	
**Physician** **satisfaction with patient-physician interaction (physician-sided PSQ)**				.08
	Descriptive, mean (SD); n	76.3 (14.9); 203	73.7 (15.3); 191	
	Estimate for the fixed effect of the study group in the linear mixed model (intervention to control group), 95% CI	N/A	–2.7 (–5.4 to 0.5)	

^a^Patient anxiety was assessed at baseline, after using the SCA (intervention group only), and after the physician encounter using a visual analog scale ranging from 0 to 100, with lower values indicating less anxiety. We conducted a multicenter, controlled parallel-group trial at 3 study sites in Berlin, Germany. Between April 2022 and January 2023, we recruited self-referred adult walk-in patients presenting with an acute, undiagnosed chief complaint. The intervention group self-assessed their complaints using an SCA before their first encounter with a treating physician, while the control group received care as usual. After the patient-physician encounter, we surveyed both patients and physicians regarding the patient-physician interaction and their satisfaction with care. Additionally, we assessed patients’ anxiety about their symptoms at multiple time points during the trial—before and after the physician encounter, and, in the intervention group, also after using the SCA.

^b^PSQ: Patient Satisfaction Questionnaire.

^c^N/A: not applicable.

^d^ZUF-8: Fragebogen zur Patientenzufriedenheit.

^e^SCA: symptom checker app.

### Secondary Outcomes

#### Satisfaction With Care (ZUF-8)

On average, participants in the intervention and control groups reported similar ZUF-8 average scores (control group: mean 2.6, SD 0.2, n=190; intervention group: mean 2.6, SD 0.2, n=173). The estimated group difference (0.02, 95% CI –0.02 to 0.06, *P*=.27) was lower than the hypothesized 0.125.

#### Anxiety Induced by SCA Usage

On average, patients reported slightly lower anxiety levels immediately after using the SCA (66.7 vs 64.5, ie, –1.7 points on a scale from 0 to 100, n=199), though this decrease was not statistically significant (estimate –0.1, 95% CI –5.0 to 4.5, *P*=.96). Approximately one-third of patients (70/199, 35.2%) in the intervention group reported increased anxiety after the SCA assessment, with about half of them (36/70) experiencing an increase of more than 5 points. Meanwhile, one-quarter (52/199, 26.1%) of patients in the intervention group reported a decrease in anxiety by more than 5 points. In both groups, one-fifth of participants reported higher anxiety levels after the physician encounter compared with their baseline level (see Table 2).

#### Treating Physicians’ Satisfaction With Interaction (Physician-Sided PSQ)

Participating physicians completed the physician-facing PSQ in 40 of the 434 (9.2%) cases, with scores missing for 19 out of 222 patients in the control group and 21 out of 212 patients in the intervention group. On average, physicians reported lower mean PSQ scores for patients in the intervention group (73.7, SD 15.3) compared with those in the control group (76.3, SD 14.9). This group difference was less than the 5 points (on a scale from 0 to 100) deemed relevant in the study protocol and was not statistically significant (95% CI –5.4 to 0.5, *P*=.08). In 2 of the 4 imputation methods applied, mean differences reached significance (see Table S3 in Multimedia Appendix 1). These differences remained below the predefined 5-point group difference deemed clinically relevant. For all remaining secondary outcomes, none of the 4 imputation approaches yielded statistically significant differences (see Table S3 in Multimedia Appendix 1).

Differences in primary and secondary outcome measures between the intervention and control groups did not reach statistical significance, even when analyzing only patients in the intervention group whose treating physician reviewed the SCA report (see Table S7 in Multimedia Appendix 1).

### Further Exploratory Analyses

#### Patient-Reported Effects of the SCA

When asked about the perceived effect of the SCA on the patient-physician interaction, more than half of the participants in the intervention group reported no effect (95/164, 57.9%). More participants reported a (rather) positive influence (66/164, 40.2%) than a (rather) negative effect (3/164, 1.8%). Similar results were observed regarding the perceived effect on the care received (see Table S4 in Multimedia Appendix 1). These findings remained consistent even when considering only cases where the physician indicated having reviewed the SCA report (data not shown).

#### Effects of the SCA Based on Physician-Reported Outcomes

Physicians reported having taken notice of the SCA’s summary report and recommendations for the majority of patients in the intervention group (112/187, 59.9%). Most physicians indicated that the SCA was neither helpful nor unhelpful for the 5 prespecified tasks (see Table S5 in Multimedia Appendix 1). However, for all 5 tasks, the SCA was rated as (rather) helpful more often than (rather) unhelpful. The SCA was considered most helpful for history taking, diagnosis, and conveying information to the patient. This finding remains unchanged when considering only cases in which the treating physician indicated having seen the SCA’s report (data not shown).

Physicians rated their satisfaction with the patient care they provided, the adequacy of time to diagnosis, and the overall length of patient stay as similar across both trial groups (see Table S6 in Multimedia Appendix 1). In both groups, treating physicians reported only a few instances of patients questioning their authority (intervention group: 2/188, 1.1%; control group: 4/184, 2.2%).

## Discussion

### Principal Findings

The AkuSym study was the first RCT to examine the impact of using an SCA before contact with the treating physician on patients’ satisfaction with the patient-physician interaction. SCA usage in the ED had no significant effect on the primary endpoint (patients’ satisfaction with the patient-physician interaction) or the prespecified secondary endpoints (patients’ anxiety, patients’ satisfaction with care, and physicians’ satisfaction with the patient-physician interaction). Neither patients’ nor physicians’ satisfaction with their interaction increased with patients’ prior use of the SCA, nor did patients’ satisfaction with the care they received or physicians’ satisfaction with the care they provided. Similarly, measures related to care efficiency, such as physicians’ assessments of time to diagnosis and length of stay, showed no benefit of the SCA, with no differences between the treatment groups. This contrasts with the perceived effects of the SCA, as about one-third of patients in the intervention group reported that it had a positive impact on their patient-physician interaction and the care they received.

This discrepancy between the measured effects of the SCA and patients’ perceptions aligns with previous literature on patients seeking health information online before receiving urgent care services. A survey-based observational study suggested a positive impact, with 150 out of 196 (76.5%) patients who had searched for information on their health problems before visiting an ED reporting that it improved the patient-physician relationship [5]. Meanwhile, an RCT primarily investigating the effect of web-based searches on the accuracy of patient-generated differential diagnoses found no evidence of an effect on its secondary endpoints, including patient-reported satisfaction with care, patients’ and physicians’ satisfaction with the patient-physician relationship, and patient anxiety [4].

We observed the same discrepancy between the perceived and measurable influence of the SCA among treating physicians. While physicians in the intervention group often considered their patients’ use of the SCA helpful for certain tasks, such as diagnosis, this did not translate into a meaningful difference in their appraisal of the adequacy of time to diagnosis between the intervention and control groups.

These discrepancies raise further questions about why the perceived positive impact did not materialize in our study. Possible explanations include limitations in our choice of endpoints, effects that emerge only in specific subgroups, or differences in usage scenarios.

Our trial does not provide evidence of benefits associated with SCAs in acute care. However, it also does not indicate any negative effects, which are often the primary concern among providers and patients. More patients reported a significant decrease rather than an increase in anxiety after using the SCA, and only a few physicians noted instances of patients questioning their authority, with no difference between the groups.

As many as 2 in 5 physicians did not review the SCA report before seeing their patient, which may have diminished its impact on the patient-physician interaction. Allowing patients to bring up the report themselves and leaving it to the physician’s discretion to consult a decision support or documentation tool more accurately reflects the reality of acute care. Therefore, we deliberately chose not to recommend that physicians engage with the SCA report.

This study has limitations. SCAs are used in various scenarios by users with diverse expectations [1,7,17,49], and our findings may not be generalizable to all these contexts. In our trial, patients used the SCA while in an ED, whereas its effect on anxiety levels might differ if used at home or outside a health care setting. Additionally, our study included only one of many available SCAs, and different SCAs may influence patients, physicians, and clinical care in distinct ways. We deliberately focused on investigating the SCA’s effects on the patient-physician relationship and satisfaction with care. Our study does not evaluate the investigated SCA’s utility in guiding patients safely and efficiently through the health care system, as we recruited patients who had already decided to seek care at 1 of the 3 trial sites. Consequently, the participating patients’ assessment of the SCA’s utility does not reflect its potential value in the context of consulting the SCA before seeking care.

Furthermore, our findings may not capture important differences between subgroups. The positive or negative effects of SCAs might be specific to certain users, such as those with prior experience with online health information or individuals with hypochondriac traits. Notably, most patients in the intervention group had never used an SCA before. This could be a strength of our trial, as it allows us to estimate the effects of SCAs when used by a broader and more diverse population beyond early adopters. The impact of SCA use may differ between current users who independently choose to use these apps and those who are prompted to do so by health care providers. However, the proportion of trial participants with prior experience using SCAs was too small in our sample to allow for meaningful analysis. Additionally, all trial sites were located in highly urban areas, and the study participants were younger on average than the overall patient population presenting at these sites during the recruitment period. As a result, our patient sample is not fully representative of the broader acute care patient population.

Because of German employee data protection regulations, we were not permitted to match participating patients with their treating physicians. As a result, we are unable to assess whether physician-related variables may have confounded our results.

Postrecruitment, we observed a baseline age difference, with control group participants being, on average, 5 years older than those in the intervention group. Despite rigorous efforts, we found no violations of the recruitment procedure that could explain this discrepancy. As younger age correlated with higher usability ratings in our study, the younger average age of the intervention group may have biased our results in favor of the SCA.

While research continues to explore differences between search engine–based access to online health information and SCAs, advances in generative artificial intelligence have introduced new tools for both laypersons and professionals. Although some SCAs, including the one used in this trial, have demonstrated high usability, accuracy, and safety, our findings suggest that these attributes alone may not translate into added value for clinical care. This perspective is supported by a recent study that found no improvement in diagnostic quality when using a commercially available, physician-facing computerized diagnostic decision support system [74]. Even newer generative artificial intelligence–based apps designed to support clinical care may encounter similar challenges [75]. Possible avenues for advancement include integrating these intelligent support tools more seamlessly into existing processes or expanding their functionalities [76].

### Conclusions

In summary, to our knowledge, we conducted the first non-industry–funded randomized controlled trial investigating the clinically relevant effects of using an SCA (Ada Health) in an acute care setting. Our trial provides no evidence of meaningful positive or negative effects of SCA use before the physician encounter on the patient-physician relationship or satisfaction with care. However, both patients and physicians more often perceived the SCA’s influence as positive rather than negative. Thus, it remains an open question whether the perceived positive effects are unsubstantiated, were not captured by our chosen endpoints, or emerge only in specific subgroups or different usage scenarios. Our study did not identify negative effects of SCA use commonly described in the literature, such as inducing anxiety or eroding patients’ trust in health care professionals. Our trial highlights the need for clinical research on mobile health apps, as high usability and reported accuracy did not necessarily translate into improved patient- and physician-reported outcomes.

## Appendix

Multimedia Appendix 1 Additional analysis.

Multimedia Appendix 2 CONSORT 2010 checklist.

## Abbreviations

CCM: Charité – Universitätsmedizin Berlin, Campus Mitte in Berlin-Mitte
CONSORT: Consolidated Standards of Reporting Trials
CSQ-8: 8-item Client Satisfaction Questionnaire
CVK: Campus Virchow-Klinikum in Berlin-Wedding
ED: emergency department
JKB: Jüdisches Krankenhaus Berlin in Berlin-Gesundbrunnen
MTS: Manchester Triage System
PSQ: Patient Satisfaction Questionnaire
SCA: symptom checker app
ZUF-8: 8-item Fragebogen zur Patientenzufriedenheit

## Data Availability

Because of privacy concerns, we are unable to share the data publicly at the time of submission.
