# Supplementary material for: Impact of a Symptom Checker App on Patient-Physician Interaction Among Self-Referred Walk-In Patients in the Emergency Department: Multicenter, Parallel-Group, Randomized, Controlled Trial
Source: J Med Internet Res. 2025 Apr 2;27:e64028. doi: 10.2196/64028 (PMC12004029; doi:10.2196/64028)
Supplement: Multimedia Appendix 1 [file jmir_v27i1e64028_app1.docx]

Supplementary tables and figures

Better off with an app? Examining the impact of a symptom assessment application on patient-physician interaction among self-referred walk-in patients in the emergency department (AkuSym): a multi-center, parallel-group, randomized, controlled trial

Malte L. Schmieding, Marvin Kopka, Myrto Bolanaki, Hendrik Napierala, Maria B. Altendorf, Doreen Kuschick, Sophie K. Piper, Lennart Scatturin, Konrad Schmidt, Claudia Schorr, Alica Thissen, Cornelia Wäscher, Christoph Heintze, Martin Möckel, Felix Balzer, Anna Slagman

# Table of Contents

[Table of Contents 1](#_Toc193733250)

[Supplementary Table 1. Comparison of age and sex between included patients and all patients at study site per study site 3](#_Toc193733251)

[Supplementary Table 2. Primary and secondary outcomes per study site 5](#_Toc193733252)

[Supplementary Table 3. Sensitivity analysis: imputation of missing data for primary outcome 6](#_Toc193733253)

[Analysis without imputation 6](#_Toc193733254)

[Imputation with mean 6](#_Toc193733255)

[Predictive mean matching (PMM) imputation 6](#_Toc193733256)

[Imputation by classification and regression trees (CART) 6](#_Toc193733257)

[Imputing by direct use of least absolute shrinkage and selection operator (LASSO) linear regression 7](#_Toc193733258)

[Supplementary Table 4. Exploratory results: patients’ perceived effect of SCA usage on patient-physician interaction and care received (intervention group only) 8](#_Toc193733259)

[Supplementary Table 5. Exploratory results: physicians’ perceived helpfulness of the SCA with pre-specified tasks 9](#_Toc193733260)

[Supplementary Table 6. Comparison of physicians’ satisfaction with care they provided, time to diagnosis and patients’ length of stay between trial groups 10](#_Toc193733261)

[Supplementary Table 7. Primary and secondary endpoints according to each study group in the modified intention-to-treat population when excluding intervention participants whose physician did not indicate to have taken notice of the SCA’s output. 11](#_Toc193733262)

# Supplementary Table 1. Comparison of age and sex between included patients and all patients at study site per study site

|  | | CCM | | CVK | | JKB | |
| --- | --- | --- | --- | --- | --- | --- | --- |
|  |  | Included in trial | All patients at trial site during recruitment period | Included in trial | All patients at trial site during recruitment period | Included in trial | All patients at trial site on recruiting days |
| n |  | 259 | 32,196 | 118 | 27,439 | 57 | 94 |
| Age |  | 32 (15) | 41 (31) | 35 (21) | 44 (35) | 36 (27) | 33 (24) |
| Sex |  |  |  |  |  |  |  |
|  | Male | 108 (42%) | 16,808 (52%) | 57 (48%) | 13,919 (51%) | 23 (40%) | 38% |
|  | Female | 134 (52%) | 15,375 (48%) | 55 (47%) | 13,516 (49%) | 31 (54%) | 59% |
|  | Diverse | 4 (2%) | - | 0 (0%) | - | 0% | 0% |
|  | NA | 13 (5%) | 13 (0.04%) | 6 (5%) | 4 (0.01%) | 3 (5%) | 3% |

*Data are n/N (%) or median (IQR). CCM = Campus Charité Mitte, Charité - Universitätsmedizin Berlin. CVK = Campus Virchow-Klinikum, Charité - Universitätsmedizin Berlin. JKB = Jüdisches Krankenhaus Berlin, emergency practice run by Berlin’s association of statutory health insurance physicians. For trial site JKB only patients presenting on days of recruitment are included as the study team had no access to clinical and administrative documentation for patients presenting outside of recruitment days. NA refers to both missing responses and respondents indicating their preferences not to state their sex. At CVK and CCM, sex is encoded binary in routine documentation.*

# Supplementary Table 2. Primary and secondary outcomes per study site

|  | CCM | | CVK | | JKB | |
| --- | --- | --- | --- | --- | --- | --- |
|  | Control | Intervention | Control | Intervention | Control | Intervention |
| **Patient satisfaction with patient-physician interaction (patient-sided PSQ)** | | | | | | |
| Mean (SD) | 81.3 (19.2); n=116 | 82.0 (17.0); n=107 | 78.0 (20.4); n=47 | 66.5 (22.9); n=40 | 83.2 (20.2); n=27 | 82.2 (20.6); n=26 |
| **Patient satisfaction with care (ZUF-8)** | | | | | | |
| Mean (SD) | 2.6 (0.2); n=116 | 2.6 (0.2); n=107 | 2.6 (0.2); n=47 | 2.6 (0.2); n=40 | 2.5 (0.2); n=27 | 2.6 (0.2); n=26 |
| **Change in anxiety level, before SCA use to after** | | | | | | |
| Mean (SD) | - | -3.9 (15.2); n=121 | - | 0.1 (9.8); n=51 | - | 4.6 (8.9); n=27 |
| **Participants more anxious after the physician encounter than at baseline** | | | | | | |
| n (%) | 23/117 (19.7%) | 20/107 (18.7%) | 8/47 (17%) | 7/40 (17.5%) | 8/27 (29.6%) | 9/26 (34.6%) |
| **Physician** **satisfaction with patient-physician interaction (physician-sided PSQ)** | | | | | | |
| Mean (SD) | 75.7 (15.4); n=124 | 74.6 (15.3); n=118 | 76.7 (12.9); n=52 | 72 (13.9); n=45 | 78.8 (16.2); n=27 | 73 (17.4); n=28 |

# Supplementary Table 3. Sensitivity analysis: imputation of missing data for primary outcome

## **Analysis without imputation**

| Endpoint | Mean Difference | Estimates (95% CI) | *P* value |
| --- | --- | --- | --- |
| Patient-sided PSQ Control vs· Intervention | -2·3 | -2·4 (-6·3 to 1·1) | 0·237 |
| ZUF-8 Control vs Intervention | 0·0 | 0·02 (-0·02 to 0·06) | 0·271 |
| Anxiety Pre vs. Post SCA use | -1·7 | -0·1 (-5·0 to 4·5) | 0·961 |
| Physician-sided PSQ Control vs· Intervention | -2·6 | -2·7 (-5·49 to 0·5) | 0·084 |

## **Imputation with mean**

| Endpoint | Mean Difference | Estimates (95% CI) | *P* value |
| --- | --- | --- | --- |
| Patient-sided PSQ Control vs. Intervention | -1·92 | -2·01 (-5·82 to 1·4) | 0·243 |
| ZUF-8 Control vs. Intervention | 0·02 | 0·02 (-0·01 to 0·05) | 0·271 |
| Anxiety Pre vs. Post SCA use | -2·07 | -0·47 (-5·34 to 3·04) | 0·636 |
| Physician-sided PSQ Control vs· Intervention | -2·38 | -2·38 (-5·33 to 0·31) | 0·084 |

## **Predictive mean matching (PMM) imputation**

| Endpoint | Mean Difference | Estimates (95% CI) | *P* value |
| --- | --- | --- | --- |
| Patient-sided PSQ Control vs. Intervention | -2·29 | -2·37 (-6·4 to 1·25) | 0·194 |
| ZUF-8 Control vs. Intervention | 0·01 | 0·01 (-0·02 to 0·04) | 0·572 |
| Anxiety Pre vs. Post SCA use | -2·75 | -0·71 (-6·44 to 2·87) | 0·480 |
| Physician-sided PSQ Control vs· Intervention | -2·95 | -2·95 (-6·02 to -0·13) | 0·041 |

## **Imputation by classification and regression trees (CART)**

| Endpoint | Mean Difference | Estimates (95% CI) | *P* value |
| --- | --- | --- | --- |
| Patient-sided PSQ Control vs. Intervention | -0·98 | -1·06 (-5·16 to 2·62) | 0·567 |
| ZUF-8 Control vs. Intervention | 0·01 | 0·01 (-0·02 to 0·04) | 0·591 |
| Anxiety Pre vs. Post SCA use | -1·81 | -0·19 (-5·56 to 4·03) | 0·846 |
| Physician-sided PSQ Control vs· Intervention | -2·58 | -2·58 (-5·63 to 0·21) | 0·070 |

## **Imputing by direct use of least absolute shrinkage and selection operator (LASSO) linear regression**

| Endpoint | Mean Difference | Estimates (95% CI) | *P* value |
| --- | --- | --- | --- |
| Patient-sided PSQ Control vs. Intervention | -2·26 | -2·35 (-6·53 to 1·4) | 0·214 |
| ZUF-8 Control vs. Intervention | 0·03 | 0·03 (0 to 0·06 | 0·073 |
| Anxiety Pre vs. Post SCA use | -2·24 | -0·79 (-5·59 to 2·24) | 0·428 |
| Physician-sided PSQ Control vs· Intervention | -3·79 | -3·79 (-6·91 to -0·93) | 0·010 |

*Mean differences, estimates, 95%-CI and p-values of primary and secondary outcomes on original and various imputed datasets. PSQ = Patient Satisfaction Questionnaire. ZUF-8 = Fragebogen zur Patientenzufriedenheit, a German version of the Client Satisfaction Questionnaire (CSQ-8). SCA = symptom checker app. For PMM, CART, and LASSO, age and sex (dummy-coded as binary; for imputation purposes, participants with diverse sex were treated as patients with missing sex indication due to their small number) were used as predictors. Imputations were performed using the R package mice (Version 3.16.0). The datasets completed via imputation were analysed in the same way as the original dataset described in section Statistical analyses.*

# Supplementary Table 4. Exploratory results: patients’ perceived effect of SCA usage on patient-physician interaction and care received (intervention group only)

|  | Negative | Rather Negative | None | Rather Positive | Positive |
| --- | --- | --- | --- | --- | --- |
| Patient-physician-interaction | 1/164 (<1%) | 2/164 (1%) | 95/164 (57.9%) | 39/164 (23.8%) | 27/164 (16.5%) |
| Care received | 1/162 (<1%) | 3/162 (2%) | 90/162 (55.6%) | 46/162 (28.4%) | 22/162 (13.6%) |

*Data are n/N (%). Single choice questions. Missing values are omitted. Intervention group only.*

# Supplementary Table 5. Exploratory results: physicians’ perceived helpfulness of the SCA with pre-specified tasks

|  | (Rather) Helpful | Neither nor | (Rather) Unhelpful |
| --- | --- | --- | --- |
| Anamnesis | 76/186 (40.9%) | 99/186 (53.1%) | 11/186 (5.9%) |
| Diagnosis | 68/187 (36.4%) | 106/187 (56.7%) | 13/187 (7%) |
| Therapy | 28/185 (15.1%) | 142/185 (76.8%) | 15/185 (8.1%) |
| Documentation | 55/185 (29.7%) | 120/185 (64.9%) | 10/185 (5.4%) |
| Conveying information to the patient | 66/184 (35.9%) | 103/184  (56.0%) | 15/184  (8.2%) |

*Data are n/N (%). Single choice questions. Missing values are omitted. Control group only.*

# Supplementary Table 6. Comparison of physicians’ satisfaction with care they provided, time to diagnosis and patients’ length of stay between trial groups

|  | Control | Intervention |
| --- | --- | --- |
| Satisfaction with care provided | 52.8 (23.6); n=202 | 51.4 (20.5); n=186 |
| Adequacy of time to diagnosis | 54.2 (25); n=201 | 51.4 (23.6); n=186 |
| Adequacy of patient’s length of stay | 52.8 (23.6); n=199 | 51.4 (18.1); n=185 |

*Data are median (IQR). Scales range from 0 – 100 for all three items, higher values indicate greater satisfaction with care provided, higher adequacy of time to diagnosis or higher adequacy of a trial patient’s length of stay, respectively.*

# Supplementary Table 7. Primary and secondary endpoints according to each study group in the modified intention-to-treat population when excluding intervention participants whose physician did not indicate to have taken notice of the SCA’s output.

| Endpoints | | Control | Intervention | *P* value |
| --- | --- | --- | --- | --- |
| **Patient satisfaction with patient-physician interaction (patient-sided PSQ^a^)** | |  |  | .31 |
|  | Descriptive, mean (SD); n | 80.8 (19.6); 190 | 78.5 (19.6); 98 |  |
|  | Estimate for the fixed effect of the study group in the linear mixed model (intervention to control group), 95% CI | N/A | –2.4 (–7.2 to 2.3) |  |
| **Patient satisfaction with care (ZUF-8^b^)** | |  |  | .99 |
|  | Descriptive, mean (SD); n | 2.6 (0.2); 190 | 2.6 (0.2); 98 |  |
|  | Estimate for the fixed effect of the study group in the linear mixed model (intervention to control group), 95% CI | N/A | 0.0003 (–0.05 to 0.04) |  |
| **Change in anxiety level, before SCA^c^ use to after** | |  |  | .71 |
|  | Descriptive, mean (SD); n | N/A | –0.7 (12.7); 110 |  |
|  | Estimate for the fixed effect of the study group in the linear mixed model, 95% CI | N/A | 1.0 (–4.6 to 6.9) |  |
| **Participants more anxious after the physician encounter than at baseline** | |  |  | .69 |
|  | n/N (%) | 39/191 (20.4) | 22/98 (22.4) |  |
|  | Estimate for the fixed effect of the study group in the generalized linear mixed model, 95% CI | N/A | 0.1 (–0.6 to 0.7) |  |
| **Physician** **satisfaction with patient-physician interaction (physician-sided PSQ)** | |  |  | .26 |
|  | Descriptive, mean (SD); n | 76.3 (14.9); 203 | 74.4 (14.8); 112 |  |
|  | Estimate for the fixed effect of the study group in the linear mixed model (intervention to control group), 95% CI |  | –1.9 (–5.8 to 1.5) |  |
